# Role of Extracellular Vesicles in Placental Inflammation and Local Immune Balance

**DOI:** 10.1155/2021/5558048

**Published:** 2021-06-18

**Authors:** Zengfang Wang, Ruizhen Yang, Jiaojiao Zhang, Pingping Wang, Zengyan Wang, Jian Gao, Xue Liu

**Affiliations:** ^1^Department of Gynecology and Obstetrics, Maternal and Child Health Hospital of Weifang Medical University, Weifang 261000, China; ^2^Operating Room, Zhucheng People's Hospital, Zhucheng 262200, China; ^3^Central Laboratory of Weifang People's Hospital, Weifang 261000, China; ^4^Department of Paediatrics, Maternal and Child Health Care Hospital of Weifang, Weifang 261000, China

## Abstract

**Background:**

Pregnancy maintenance depends on the formation of normal placentas accompanied by trophoblast invasion and vascular remodeling. Various types of cells, such as trophoblasts, endothelial cells, immune cells, mesenchymal stem cells (MSCs), and adipocytes, mediate cell-to-cell interactions through soluble factors to maintain normal placental development. Extracellular vesicles (EVs) are diverse nanosized to microsized membrane-bound particles released from various cells. EVs contain tens to thousands of different RNA, proteins, small molecules, DNA fragments, and bioactive lipids. EV-derived microRNAs (miRNAs) and proteins regulate inflammation and trophoblast invasion in the placental microenvironment. Maternal-fetal communication through EV can regulate the key signaling pathways involved in pregnancy maintenance, from implantation to immune regulation. Therefore, EVs and the encapsulating factors play important roles in pregnancy, some of which might be potential biomarkers.

**Conclusion:**

In this review, we have summarized published studies about the EVs in the placentation and pregnancy-related diseases. By summarizing the role of EVs and their delivering active molecules in pregnancy-related diseases, it provides novel insight into the diagnosis and treatment of diseases.

## 1. Introduction

**Figure 1:**
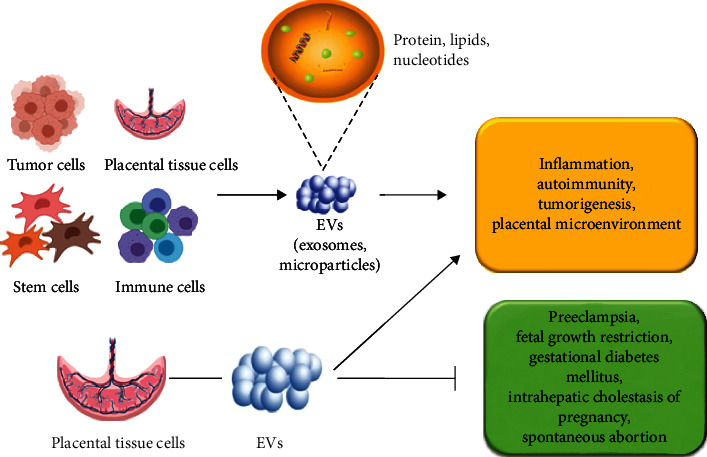
Role of EVs in placenta and pregnancy disorders. EVs mainly include exosomes and particles, which can be derived from stem cells, tumor cells, immune cells, and placental tissue cells. They participate in intercellular communication by transmitting bioactive proteins, lipids, and nucleotides to targeted cells. EVs, especially EVs derived from placental tissue cells, play a crucial role in regulating inflammation, autoimmune, tumor occurrence, and placental microenvironment balance. EVs have been used to prevent pregnancy diseases such as PE, fetal growth restriction, and spontaneous abortion.

Extracellular vesicles (EVs) are secreted by cell membrane or cells with diameters ranging from 40 nm to 1000 nm [1, 2]. EVs mainly include microbubbles, ectosomes, exosomes, and microparticles according to their subcellular sources [3–5]. Exosomes with a diameter of 40~100 nm are the most commonly studied EVs, which are firstly found in sheep reticulocytes [6]. EV plays an important role in a series of biological processes by transferring lipids, proteins, nucleotides, and other bioactive components. These encapsulated bioactive molecules can be delivered to specific targeted cells (Figure 1). In particular, some EVs carrying noncoding RNAs, such as microRNA (miRNA) and long-chain noncoding RNAs (lncRNA), can be transferred to specific cells and regulate the expression and function of target mRNA at different biological stages [7]. EVs participate in the exchange of substances and information between cells mainly through the following pathways [8–10]. Firstly, EV membrane proteins can bind to the targeted cell membrane, thereby activating the signaling pathway in cells. Secondly, EVs can be digested by proteases in extracellular matrix. The digested fragments can be used as ligands to bind to the receptors on the cell membrane, thereby regulating intracellular communication. Thirdly, EVs can be directly fused with the target cell membrane, and then, EVs containing bioactive components are nonselectively released into recipient cells.

EVs from different cell sources can exert multiple effects. EVs derived from mesenchymal stem cells (MSC) can significantly improve myocardial cell survival, prevent myocardial cell injury, promote angiogenesis, and improve cardiac function by regulating inflammation and autoimmune [11–13]. Due to the advantages of nanomolecular structure and excellent biocompatibility, EVs have great application potential as drug carriers. MSC-derived EVs are used to treat chronic skin ulcers, which can promote wound healing and reduce scar formation [14]. In addition, EVs play a key role in tumor occurrence, immune surveillance, immune escape, and tumor microenvironment reprogramming [15–17]. Accordingly, EVs and their encapsulated bioactive molecules are essential for intercellular communication in many diseases, including pregnancy disorders. The role of EVs in immune regulation has been extensively studied. EVs from intestinal epithelial cells (IECs) released during sepsis have been reported to improve intestinal inflammation by transferring miRNAs into cells [18]. EVs are involved in maintaining T cell tolerance and long-term T cell memory [19]. EV-encapsulated proteins participate in immune regulation by influencing the secretion of anti-inflammatory cytokines [20]. Thyroid stimulating hormone receptor (TSHR) released by EV improves autoantibody-mediated activation of Graves disease by blocking autoantibodies [21]. Baskaran et al. reported that EVs play an indispensable role in sperm maturation by regulating autoimmunity [22]. This local immunity privilege at the maternal-fetal interface has been attributed to the expression of Fas ligand (FasL) related to placental exosomes, programmed death ligand 1 (PD-L1), and TNF-related apoptosis-inducing ligand (TRAIL), which all induce maternal T cell incompetence and death [23, 24]. Expression of NKG2D receptor ligand, UL-16-binding protein (ULBP), and MHC class I chain-related protein (MIC) on placental exosomes have been shown to downregulate NK cell activity and inhibit maternal cytotoxicity [25].

In the past decades, the role of EV in regulating placenta implantation and pregnancy diseases has attracted much attention due to its key role in regulating inflammation and immunity. The aim of this study is to summarize the current published studies on the association of EVs with placenta implantation and pregnancy-related diseases. In particular, we aim to explore the role of EVs in the diagnosis and treatment of pregnancy-related diseases.

## 2. EVS from Placental Tissues and Stem Cells

Placenta is the fundamental physiological barrier for maternal immune tolerance to fetus and various pathogens. EVs released by syncytiotrophoblasts, trophoblasts, extravillous trophoblasts, and placental vascular endothelial cells play a key role in pregnancy [26]. Placenta and umbilical cord are rich in MSCs, which are promising treatment strategies for various diseases [27]. It has been shown that EVs from human umbilical cord MSCs (hUC-MSCs) could protect against severe burn-induced hyperinflammation through the way of paracrine secretion [28]. Surico et al. have found that hUC-MSCs are involved in preeclampsia (PE) and affect fetal growth restriction (FGR) by delivering bioactive components encapsulated in EVs [29]. hUC-MSC-derived EVs have also been found to confer effect on the paracrine of trophoblast cells, which facilitates their use in treating pregnancy-related disorders. Decidual MSC-derived EVs can also exert inhibitory effects on angiogenesis and maintain the balance of maternal-fetal interface by regulating macrophage polarization in PE [30]. Accordingly, placenta tissue MSCs and hUC-MSC-derived EVs play key roles in maintaining placental functions and protecting against pregnancy disorders.

Accumulated data have shown that MSC transplantation may be extremely beneficial for pregnancy-associated diseases [31]. In the past few years, the use of MSC-EVs in pregnancy disorders has drawn more and more attention. Placental tissue cell-derived EVs can be produced regardless of physiological or pathological pregnancy. Reduced circulating EVs are associated with placental dysfunction [32]. Circulating EVs play a crucial role during pregnancy [33]. EVs derived from placental tissue cells increased gradually with the progress of pregnancy, especially during delivery, but significantly decreased to nonpregnancy level 48 hours after delivery [34]. EVs derived from placental tissue cells can selectively transfer bioactive molecules, such as stress-induced protein molecules, apoptosis-related molecules, cytokines, mRNA, and miRNA. EVs actively participate in maternal-fetal communication during pregnancy by regulating different processes [35]. The fetus is the mother's allogeneic graft. Successful pregnancy depends on the maintenance of maternal-fetal interface immune tolerance. Th1/Th2 balance during pregnancy is essential to maintain maternal and fetal microenvironment [36]. Placenta-derived EVs promote maternal immune tolerance by inhibiting maternal T cell response and maintaining Th1/Th2 balance [35]. Placenta-derived EVs produce Syn-2, which proves to play an immunosuppressive role in placenta [37]. Placental tissue-derived EVs and exosome inclusion factors are important regulators of placental immunity and homeostasis [37]. The delivery of bioactive molecules by EVs is beneficial to placental function. During pregnancy, EVs can be transported to the fetal side as drugs and other goods [38]. EVs also play an important role in regulating systemic inflammation by releasing cytokines. Therefore, EVs from placenta tissues and stem cells are essential for maintaining maternal-fetal immunity and protecting inflammation.

MSC-derived noncoding RNAs can serve as useful biomarkers in pregnancy disorders [28–30]. However, whether MSC-derived noncoding RNAs can exert their effects through EVs and mediate intercellular communications in maternal-fetal immunity needs to be elucidated in the future. During the past few decades, noncoding RNAs derived from placental stem cells have been demonstrated to participate in pregnancy regulation, such as lncRNAs and miRNAs [39–41]. Placental tissue-derived EV miRNAs have been implicated in the antiviral immunity at the maternal and fetal interface [42]. In addition, studies have found that EVs from placental tissue cells also have significant effects on pregnancy complications by transferring miRNAs to recipient cells [43]. Accordingly, those noncoding RNAs from placental tissues and stem cell-derived EVs serve as key biomarkers for gestational disorders.

## 3. EVS and Pregnant Disorders

EVs are the “fingerprints” of their primitive cells and are involved in regulating pregnancy complications. EVs derived from placental tissue may be another way for the fetus to communicate with mothers. EV regulates inflammatory cascade reactions in some complex pregnancy, such as PE and intrauterine growth restriction [44]. Here, we summarize the research progress of the role of EVs and their encapsulated factors in some common pregnancy diseases (Table 1).

### 3.1. EVS and PE

PE is an idiopathic pregnancy disease characterized by hypertension and proteinuria after 20 weeks of pregnancy. The global incidence of PE is 3-8%, and its pathogenesis is still unclear. The basic pathological changes of PE are systemic vasospasm, vascular endothelial injury, and ischemia. PE leads to reduced perfusion of all organs and poses risks to both mothers and children. Its performance is often significantly improved after childbirth or termination of pregnancy, suggesting that placental-derived media play a key role in the pathogenesis of PE.

A number of studies have suggested EVs and their encapsulated factors are involved in the pathogenesis of PE [45, 46]. Bioactive molecules in placental tissue-derived EVs might be as potential biomarkers for the diagnosis and prognosis of PE [47]. Placental protein 13 (PP13) has been identified in maternal circulating exosomes or microvesicles [48]. Low serum level of PP13 predicts high risk of PE and other obstetric complication [48]. Salomon et al. have found that total plasma exosomes and placental tissue exosomes both significantly increased in patients with PE compared with normal pregnancies [49]. Similarly, placental EVs in the blood circulation of PE patients are significantly lower than that in patients with early-onset PE [50]. Chang et al. showed that exosome soluble fms-like tyrosine kinase-1 (sFlt-1) and soluble endoglin (sEng) inhibited the growth and tube formation of human umbilical vein endothelial cells in PE [51]. In addition, circulating EVs from placenta can activate immune cells and may affect the production of inflammatory cytokines in PE pregnancy [52]. As a result, EV-delivering factors are essential regulators in PE.

In the last decade, EV-encapsulated noncoding RNAs have been found to affect the development of PE [40]. Some placental tissue-derived miRNAs can be released into the maternal circulation from the trophoblast layer via EVs [53]. A few EV-encapsulated miRNAs are potential markers for PE [54]. Diverse miRNA profiles in circulating EVs from early and late onset of PE have been demonstrated, which implicates that EV-derived miRNAs are key factors associated with PE [55]. Li et al. have demonstrated there are specific miRNA expression and signals in serum exosomes from PE pregnancies, suggesting the modifying effect of plasma exosomal miRNAs in the pathogenesis of PE [56]. Increased expression of miR-201-3p has been demonstrated in the circulating EVs of patients with PE [57]. The study by Shen et al. has suggested that placental tissue cell-derived exosomal miR-155 inhibits the expression of endothelial nitric oxide synthase in PE [58]. In addition, there are many other EV-derived miRNAs demonstrated as key biomarkers in PE, such as EV-encapsulated miR-136 and miR-495 [59]. Exosomal miR-210 has been reported to be produced by trophoblasts and participates in the intercellular communication in PE [60]. Some EV-derived miRNAs have been suggested as circulating markers for PE, including miR-517-5p, miR-520a-5p, and miR-525-5p [61]. Accordingly, EV-encapsulated noncoding RNAs are key regulators in PE.

The role of stem cell EV-derived miRNAs in PE has also been extensively investigated in the past few years. It has been reported that the umbilical cord blood stem cells EV-derived miR-125a-5p plays a critical role in PE [62]. It has been demonstrated that hUC-MSC EVs can inhibit placental cell apoptosis and promote angiogenesis in PE [63]. Besides, MSC-derived exosomal lncRNA H19 has been demonstrated to increase the invasion and migration of trophoblast cells by regulating let-7b through AKT signaling pathway in PE [64]. Accordingly, stem cell-derived EVs and the encapsulated bioactive molecules can regulate angiogenesis and autoimmune balance in PE, which may be promising therapeutic strategy for patients with PE.

### 3.2. EVS and Gestational Diabetes Mellitus

Pregnancy can cause diabetes in pregnant women who have no diabetes previously and also exacerbate the condition of patients with existing diabetes. Gestational diabetes has a greater impact on both mothers and children, and the near-term and long-term complications of mothers and children are higher. Pregnant women with diabetes are more likely to subject to hypertension during pregnancy than nondiabetic women [65]. Diabetes can increase the chance of infection, dystocia, birth canal injury, surgery, and postpartum bleeding in pregnant women as well as elevated incidence of large children, restricted fetal growth, spontaneous abortion, embryonic termination, and premature birth.

The development of gestational diabetes mellitus is related to heredity, autoimmunity, and environmental factors. Increasing evidence has suggested the crucial role of EVs in gestational diabetes mellitus. The study by Arias et al. has demonstrated the potential role of plasma EV-encapsulated factors as early biomarkers for gestational diabetes mellitus [65]. Sharma et al. have found that human urine-derived EVs contained gluconeogenic enzymes that affect glucose metabolism [66]. Elevated phosphoenolpyruvate carboxykinase (PEPCK) in urine exosomes is related to high risk of diabetes and early insulin resistance [66]. In addition, currently available data has suggested EVs play an important role in autoimmune reaction in islets of type 1 diabetes [67]. Moreover, EVs produced by adipose tissue and muscle tissue regulate glucose and lipid balance and even the inflammatory environment by transferring specific molecules to a variety of insulin-sensitive peripheral tissues [67]. As a result, the effect of EVs in gestational diabetes mellitus is essential.

EV-encapsulated factors can be used as useful biomarkers for a variety of diseases and complications [68]. Many studies have shown EV-encapsulated proteins, mRNAs, and DNAs and are new diagnostic markers for diabetic nephropathy [68]. EVs can also be used as delivery vehicles for the treatment of diabetic nephropathy. A previous study has demonstrated that the urinary exosomal Elf3 may be an early noninvasive marker for podocyte injuries in diabetic nephropathy [69]. Mammalian sterile 20-like kinase 1- (Mst1-) enriched EVs released from cardiac microvascular endothelial cells play an important role in inhibiting autophagy, promoting apoptosis, and suppressing glucose metabolism in cardiomyocytes [70]. All these findings have supported the vital role of EVs in glucose metabolism and diabetes progression.

Insufficient myocardial angiogenesis can induce diabetes-related ischemic cardiovascular [71]. CD133^+^ exosomes derived from human umbilical cord blood improve cardiac function in diabetic stroke mice [72]. MSC EVs protect *β* cells from hypoxia-induced apoptosis [73]. More and more evidence suggests that EVs and delivered miRNAs have neuroprotective effects and may be a treatment for diabetes-related stroke [74]. Studies have shown that EVs extracted from plasma of pregnant women with gestational diabetes significantly promote the production of inflammatory cytokines [75]. EVs can also regulate placental and fetal membrane endothelial dysfunction in gestational diabetes mellitus [76]. Adipose tissue-derived EVs mediate placental immunity in gestational diabetes mellitus, which may be associated with some adverse outcomes, including fetal overgrowth [77]. EVs represent a new mechanism for regulating maternal glucose homeostasis during pregnancy [78]. Taken together, the role of EVs in gestational diabetes mellitus is critical. At the same time, the mechanism of EVs wrapped bioactive factors in gestational diabetes remains to be further studied.

Increasing evidence has supported that EV-transferred miRNAs and other noncoding RNAs can be used as promising biomarkers for gestational diseases including gestational diabetes mellitus [79]. Nair et al. have reported that placental tissue-derived EV miRNAs can regulate skeletal muscle insulin sensitivity [80]. Placental tissue-derived EVs may exert effects on insulin sensitivity in normal and gestational diabetes mellitus pregnancies. Taken together, EVs offer new options for the treatment of gestational diabetes mellitus [81]. EV-encapsulated noncoding RNAs can also serve as biomarkers for the diagnosis and treatment of gestational diabetes mellitus [82].

### 3.3. EVS and Fetal Growth Restriction (FGR)

FGR usually leads to low birthweight births. However, the underlying biological mechanism has not been fully elucidated. FGR can be secondary to various pregnancy complications, such as gestational hypertension, diabetes, and intrahepatic cholestasis. Previously found aberrantly expressed miRNAs in maternal circulation [83]. New evidence suggests that miRNAs are specifically expressed in maternal blood of FGR in the second trimester of pregnancy, suggesting possible fetal growth markers [84]. Besides, Miranda et al. reported that placental exosome-derived miRNAs in maternal plasma can predict fetal growth and can be used as indicators of placental function [85]. Studies have shown that EV miR-150 derived from umbilical vein of normal piglets can promote angiogenesis in intrauterine growth restriction pigs [86]. It is reported that circulating plasma exosomes in early pregnancy had the same C19MC microRNA as placental tissues of GH, PE, and FGR patients after delivery [87]. Maternal plasma exosome analysis of selected C19MC microRNAs [88] showed that FGR women had a new downregulated biomarker (miR-520a-5p) in the first three months of pregnancy, which was not found in maternal plasma analysis [89]. In summary, these established findings strongly demonstrate the important role of EVs and encapsulation factors including miRNAs in FGR, providing new clues for understanding the pathogenesis of FGR.

### 3.4. EVS and Intrahepatic Cholestasis of Pregnancy (ICP)

ICP is a common complication during pregnancy. Due to bile acid toxicity, the incidence and mortality of perinatal complications increased significantly. The etiology of ICP is still unclear, which may be related to female hormones, genetic and environmental factors. The previously published data show the pivotal role of EVs in ICP. Those active factors usually consist of lipids, proteins, and nucleotides. The upregulation of miR-21, miR-29a, and miR-590-3p in urinary exosomes can increase the incidence of ICP by downregulating intercellular adhesion molecule 1 (ICAM1) [90]. Nevertheless, more studies are needed to further clarify the role of EVs and EV-encapsulated bioactive factors in ICP.

### 3.5. EVS and Spontaneous Abortion or Premature Delivery

Spontaneous abortion is highly correlated with embryo chromosomal abnormalities. Premature birth will cause great harm to pregnant women and fetuses. The organ development of premature infants is not good. The smaller the gestational age at birth, the lighter the weight, and the worse the prognosis. The causes of premature delivery include infection, malnutrition, cervical insufficiency, uterine malformation, and pregnancy complications. EVs have been shown to be involved in the development of spontaneous abortion and premature delivery by regulating homeostasis imbalances, particularly inflammation and endocrine signals [91]. MSC-derived exosomes regulate maternal-fetal interface T cells and macrophage-mediated immune response, affecting the outcome of spontaneous abortion or premature delivery [92]. The key role of stem cell-derived EVs in animal models of pregnancy has also been demonstrated [93]. The exact mechanism of endometrial cell-derived EVs mediating maternal crosstalk and affecting pregnancy outcomes during implantation remains unclear. Some studies have shown that EVs derived from placental tissue can predict pregnancy outcomes, including spontaneous abortion and premature delivery. Panfoli et al. confirmed that MSC-EVs affect aerobic metabolism in term and preterm infants [94]. Most importantly, EVs are professional carriers of fetal signals that affect pregnancy outcomes [95]. Amniotic fluid EVs have been shown to deliver information to normal and abnormal births [96]. In addition, exosomal miRNAs were found in maternal circulation, which may represent the “fingerprint” of pregnancy progression [97]. In summary, the study of EVs and their bioactive factors in spontaneous abortion or premature delivery is expected to find new therapeutic strategies for these diseases.

### 3.6. EVS and Prenatal Diagnosis

Prenatal diagnosis includes chromosomal abnormalities, sexually related genetic diseases, genetic metabolic defects, and congenital structural abnormalities. The important role of circulating EVs has been demonstrated in prenatal and neurodegenerative diseases [98]. Studies have shown that EV miRNAs can be used as biomarkers for prenatal diagnosis of congenital hydronephrosis [99]. They found that reduced expression of EV-transmitting miR-300 and miR-299-5p in amniotic fluid of congenital hydronephrosis could predict renal fibrosis. Interestingly, studies by Goetzl et al. showed that EVs derived from fetal central nervous system could be purified from maternal plasma, which was related to the abnormal proliferation and differentiation of neural stem cells [100]. Therefore, EVs may be a potential way for early prenatal diagnosis of fetal neurological diseases. EVs, especially exosomes, are also involved in Down syndrome [101], suggesting that EVs play an important role in the regulation of central nervous system development. Although EVs and the transfer of bioactive factors have been associated with some prenatal and neurodegenerative diseases, more studies need to explore their role in prenatal diagnosis.

## 4. Conclusion and Perspectives

At present, studies have shown that EVs play a key role in tumorigenesis, stem cell capacity maintenance, inflammation, and immune disorders. They participate in the exchange of substances and information between cells by transmitting bioactive molecules, including lipids, proteins, and nucleotides. Cellular EVs in placenta tissue have a good record in maternal circulation, thus affecting pregnancy outcomes. Targeting EVs and the encapsulated bioactive factors may be promising strategies for the treatment and prevention of pregnancy disorders. However, the molecular mechanism and function of EVs in placenta and pregnancy-related complications warrant further elucidation in the future.

## Figures and Tables

**Table 1:** EV-derived biomarkers in autoimmunity and pregnancy disorders.

Biomarker	Type of biomarker	Type of derived EVs	Diseases and pregnancy condition	Function	Reference
Thyrotropin receptor	Receptor	Exosomes	Graves' disease	Sequestering autoantibody and ameliorating autoantibody-mediated activation	[21]
UL-16-binding protein	Protein	Exosomes	Placenta during pregnancy	Downregulate NK cell activity and inhibit maternal cytotoxicity	[25]
MHC class I chain-related protein	Protein	Exosomes	Placenta during pregnancy	Downregulate NK cell activity and inhibit maternal cytotoxicity	[25]
Syncytin-2	Protein	Exosomes	Normal pregnancy	Exerting immunosuppressive effects on T cells	[37]
Placental protein 13	Protein	Exosomes or microvesicles	PE and other obstetric complications	Binding to glycosylated receptors, bringing about hemagglutination, immunoregulation and vasodilation	[48]
Soluble fms-like tyrosine kinase-1	Protein	Exosomes	PE	Attenuating the proliferation, migration, and tube formation of human umbilical vein endothelial cells	[51]
Soluble endoglin	Protein	Exosomes	PE	Inhibiting the growth and tube formation of human umbilical vein endothelial cells	[51]
MiR-201-3p	Nucleotide/noncoding RNA	Exosomes	PE	Having a role in the pathomechanism of PE	[57]
MiR-155	Nucleotide/noncoding RNA	Exosomes	PE	Inhibiting the expression of endothelial nitric oxide synthase	[58]
MiR-136 and miR-495	Nucleotide/noncoding RNA	MSC-derived exosomes	PE	Promising circulating biomarkers in early detection of PE	[59]
MiR-210	Nucleotide/noncoding RNA	Exosomes	PE	Produced by trophoblasts and participating in the intercellular communication	[60]
Phosphoenolpyruvate carboxykinase	Kinase	Urine exosomes	Gestational diabetes mellitus	Affecting insulin resistance	[66]
Sterile 20-like kinase 1	Protein	Cardiac microvascular endothelial cell-derived exosomes	Diabetes	Inhibiting autophagy, promoting apoptosis and suppressing glucose metabolism	[70]
EVs-encapsulated miRNAs	Nucleotide/noncoding RNA	EVs	Gestational diabetes mellitus	Serving as biomarkers for the diagnosis and treatment of gestational diabetes mellitus	[79–82]
MiR-150	Nucleotide/noncoding RNA	Piglet's umbilical vein-derived EVs	FGR	Promoting angiogenesis	[86]
MiR-21, miR-29a and miR-590-3p	Nucleotide/noncoding RNA	Urinary exosomes	ICP	Downregulating intercellular adhesion molecule 1	[90]
MiR-300 and miR-299-5p	Nucleotide/noncoding RNA	EVs	Congenital obstructive nephropathy	Reregulating renal fibrosis	[99]

EVs: extracellular vesicles; FGR: fetal growth restriction; ICP: intrahepatic cholestasis of pregnancy; MSCs: mesenchymal stem cells; PE: preeclampsia.
